# Environmental influences and ontogenetic differences in vertical habitat use of black marlin (*Istiompax indica*) in the southwestern Pacific

**DOI:** 10.1098/rsos.170694

**Published:** 2017-11-01

**Authors:** Samuel M. Williams, Bonnie J. Holmes, Sean R. Tracey, Julian G. Pepperell, Michael L. Domeier, Michael B. Bennett

**Affiliations:** 1 School of Biomedical Sciences, The University of Queensland, St Lucia, Queensland 4072, Australia; 2 Institute for Marine and Antarctic Studies, University of Tasmania, Private Bag 49, Hobart, Tasmania 7001, Australia; 3 Pepperell Research and Consulting Pty Ltd, P.O. Box 1475, Noosaville DC, Queensland 4566, Australia; 4 Marine Conservation Science Institute, Fallbrook, CA, USA

**Keywords:** billfish, diving behaviour, ontogeny, physiology, telemetry

## Abstract

The black marlin (*Istiompax indica*) is a highly migratory billfish that occupies waters throughout the tropical and subtropical Indo-Pacific. To characterize the vertical habitat use of *I. indica*, we examined the temperature-depth profiles collected using 102 pop-up satellite archival tags deployed off the east coast of Australia. Modelling of environmental variables revealed location, sea-surface height deviation, mixed layer depth and dissolved oxygen to all be significant predictors of vertical habitat use. Distinct differences in diel movements were observed between the size classes, with larger size classes of marlin (greater than 50 kg) undertaking predictable bounce-diving activity during daylight hours, while diving behaviour of the smallest size class occurred randomly during both day and night. Overall, larger size classes of *I. indica* were found to use an increased thermal range and spend more time in waters below 150 m than fish of smaller size classes. The differences in the diving behaviour among size classes were suggested to reflect ontogenetic differences in foraging behaviour or physiology. The findings of this study demonstrate, for the first time to our knowledge, ontogenetic differences in vertical habitat in a species of billfish, and further the understanding of pelagic fish ecophysiology in the presence of global environmental change.

## 1. Background

Billfishes of the family Istiophoridae (marlins, sailfish and spearfishes) include some of the largest and most highly migratory species on earth. However, despite their ability to transverse ocean basins, billfish movements are restricted by their physiological tolerance to certain environmental conditions [1]. Satellite tagging has provided important insights into the influence of environmental factors on the vertical habitat use of billfish. A recent review of their diving behaviour identified four physical drivers of ecology: temperature, light, oxygen and complex water mixing (e.g. fronts and eddies) [2]. These physical drivers have been shown to effect billfish physiology, whereby optimal conditions are thought to encourage the expansion of vertical habitat use [3]. Therefore, to understand how changes in global climate patterns may influence the distribution or abundance of these large pelagic predators, it is important that vertical habitat use and environmental drivers of movement are better understood.

The effect of dissolved oxygen (DO) on diving behaviour has been extensively studied in some billfish species. Comparative investigations of sailfish (*Istiophorus platypterus*) and blue marlin (*Makaira nigricans*) vertical habitat use between the oxygen-rich waters of the Atlantic and oxygen-depleted waters of the tropical Eastern Pacific show that the extent of water column use is limited at depths where DO levels are below 3.5 ml O_2_ l^−1^ [4,5]. The availability of light and conditions influencing water mixing have also been associated with billfish vertical distribution. As billfish are visual predators, the degree of light penetration through the water column influences their vertical distribution, and they are able to forage in deeper waters during the day [6]. Diel-diving behaviour has been noted for many istiophorid billfishes including striped marlin (*Kajikia audax*) in the southwestern Pacific, where tagged fish spent daylight hours foraging in deeper waters, before returning to surface waters at night [2,3,7]. Complex water mixing such as eddies or fronts have been shown to play an important role in the foraging behaviour of pelagic predators. The formation of eddies as a driver in the aggregation of prey has been identified as a factor influencing vertical habitat use in opah (*Lampris guttatus*) [8]. Complex water mixing has also been associated with shifts in species distribution and catch per unit effort (CPUE) increases for billfishes [9,10]; however, the influence of water mixing on depth use has yet to be investigated.

The distribution of temperature at depth is another physical factor commonly associated with billfish vertical habitat use because temperature has been shown to affect the cardiac function of marine teleosts [11]. Decreases in ambient water temperature reduce metabolic rate, contractibility of cardiac muscle, cardiac output and core body temperature in marine teleosts [12]. Billfish are often restricted in the amount of time they can spend in cold deep water presumably owing to the physiological costs of operating in cooler waters, often outside a species' optimal thermal window [1,4]. Comparison of vertical habitat use among various species of billfish has led to the suggestion that larger-bodied individuals make greater use of their increased thermal inertia than smaller-bodied ones, by diving deeper [2,13]. This theory derives from the observation that the larger-bodied *M. nigricans* and *K. audax* [1,7] have been found to dive deeper than the smaller-bodied *I. platypterus* and white marlin (*Kajikia albida*) [13,14]. Despite the suggestion that a species' body mass limits the amount of time spent outside of its optimal thermal range and subsequent vertical habitat use [1,2,13], there have been no quantitative analysis of such a relationship in any billfish species. In addition, the suggestion of body mass influencing depth use also does not consider the large ecological differences between billfish species or actual mass of individuals tagged in studies [2,13]. Despite the black marlin (*Istiompax indica*) being one of the largest billfishes, tagging of *I. indica* has shown the deepest dives to be less than 250 m (minimum ambient temperate of 14.5°C) [15–17], which are equivalent to the maximum depths reported for *I. platypterus* (8.7°C) and *K. albida* (12°C), which maintain a much smaller average mass [13,14]. In addition, *I. indica* have been observed to primarily occupy the upper 50–100 m of the water column and no indications of diel-diving behaviour have been reported [15–17]. To effectively evaluate the relationship between body mass and vertical habitat use, an intraspecific comparison is required to reduce the potentially large effect of variation owing to differing species' ecologies.

*Istiompax indica* is a highly migratory istiophorid billfish that occupies waters throughout the tropical and subtropical Indo-Pacific [18]. To understand movements of *I. indica*, a number of broad-scale tagging programmes have tracked the movement of fish throughout their range [19,20]. Tagging data have revealed that horizontal movement patterns vary across life stages, with juvenile and sub-adult movements presumably motivated by prey availability, while adult movements are also influenced by reproductive philopatry [20,21]. Horizontal movements have been assessed in conjunction with physical factors to determine sea-surface habitat preferences in *I. indica*. Deployment of pop-up satellite archival tags (PSATs) on *I. indica* off the east coast of Australia revealed an affinity for waters with a sea-surface temperature (SST) between 26°C and 27°C [20]. Using spatial positions derived from conventional tagging data in a species distribution model, the occurrence of juvenile *I. indica* has been strongly correlated with sea-surface height anomaly (SSHa) and chlorophyll-*a* concentrations (Chl-*a*) [9]. While considerable efforts have been made towards understanding the horizontal movements of *I. indica* in surface waters, the paucity of information regarding vertical habitat use represents a knowledge gap [2].

*Istiompax indica* is one of the most extensively tagged billfish species, yet there is little known about how they use the water column and the influence of physical factors on their diving behaviour. In this study, we investigate: (i) the variation of vertical habitat use among differing size classes of *I. indica*, and (ii) explore physical factors to better understand how they influence the depth use of *I. indica*.

## 2. Material and methods

### 2.1. Satellite tagging

A total of 102 PSATs were deployed on *I. indica* off the east coast of Australia (Queensland and New South Wales) in consecutive years from 2002 to 2014 as part of two separate tagging programmes. All tagged fish were captured using trolled baits in a rod-and-reel fishery with the assistance of recreational anglers, with 41 of the tags part of the Great Marlin Race (GMR) (https://igmr.igfa.org/Conserve/IGMR.aspx) and the remaining 61 tags being deployed by Domeier & Speare [20]. Tag attachment methods have been previously described by Domeier & Speare [20] and GMR tagging protocols by Carlisle *et al.* [22]. The PSATs deployed were manufactured either by Wildlife Computers (WC; Redmond, WA, USA), models PAT0, PAT1, PAT2, PAT3, PAT5, PAT6, MK-10 and MiniPATs, or by recovered Microwave Telemetry (MT, Columbia, MD, USA) model PTT-100 tags. Tags were programmed to record pressure (depth), temperature and light at 20 or 60 s intervals. Tags were programmed to release from fish after 120 days (*n* = 14), 180 days (*n* = 86) or 270 days (*n* = 2) in order to capture both broad-scale and high-resolution data. Archival data containing depth, temperature and geolocation (based on light levels) were received through the Argos satellite system (Service Argos, Toulouse, France). High-resolution time-series data (recorded every 7 min) were collected from WC MiniPATs via satellite, as well as from 15 physically recovered tags (data recorded every 20 or 60 s), which were downloaded directly from each tag's data archive (electronic supplementary material, table S2).

### 2.2. Data processing

All tag data were collated using R v. 3.3.2 [23]. For WC tags, percentage time at depth (PTD) and maximum depth (MD) data were aggregated daily based on summarized daily histogram and maximum depth files. To examine diel patterns of depth and temperature, only high-resolution time-series data were used. Tagged animals were separated into five size classes based on mass estimates made by anglers and charter captains at the time of capture. As females and males mature at 100 and 40 kg, respectively, it was assumed that all tagged animals that had an estimated mass over 100 kg were mature [20]. However, owing to the inability to determine the age or sex of tagged animals for all size classes, additional groupings were based on five arbitrary mass ranges that had sufficient sample sizes for downstream analysis. These were: small (20–50 kg); intermediate (51–100 kg); medium (101–250 kg); large (251–400 kg) and very large (greater than 400 kg).

### 2.3. Geolocation estimation

Daily position estimates were calculated using a state-space model accessed through the WC proprietary software, Global Position Estimator 3 (GPE3) (WC). The state-space model uses light-level, *in situ* temperature observations and their corresponding remotely sensed reference data (twilight, SST) into a diffusion-based space-state movement model to generate time-discrete gridded probability surfaces. Grids are produced at a resolution of 0.25°. To reduce the number of unrealistic positions, an estimate of maximum animal speed was input to represent the standard deviation of diffusion rate for the animal. A starting maximum speed of 200 km d^−1^ was input, based on the knowledge that *I. indica* have been recaptured to 500 km from their tagging location after 3 days (J. Pepperell 2008, unpublished data). Animal speed was then varied to ensure the geolocation estimation was within the model bounds and stabilized across runs, with optimal animal speed for all tagged *I. indica* being estimated at 250 km d^−1^.

### 2.4. Data analysis

Environmental data were derived from broad-scale climatological datasets to investigate associations with *I. indica* vertical habitat use. Monthly 1°-grid climatology data of DO at 100, 200 and 300 m depths were sourced from World Ocean Atlas 2009 (WOA09), and a suite of satellite remote-sensed data (electronic supplementary material, table S1) from National Oceanic and Atmospheric Administration (NOAA) Coastwatch were extracted along daily track positions using *xtractomatic* routines in R [23] as described by Lam *et al.* [3]. To account for error in the position estimates and ensure consistent spatial coverage of remote-sensed data, an average value for each given position was calculated with a 0.25° error. An estimate of Δ*T* was calculated as the difference between remote-sensed SST and archival tag-sampled temperatures at depth. Positive Δ*T* values reflect cooler measured temperature than SST [4].

To account for any spatial variability when investigating differences in vertical habitat use, data were sub-sampled from an area of spatial overlap based on the daily position estimates from the most probable track of tagged animals across all five size classes. The area of spatial overlap was defined by observations within latitudes of 10° S–20° S and longitudes of 145° E–160° E. The sub-sampled data were plotted to inspect the data for normality and to check that the distribution of the data was consistent among size classes. To assess for differences in the MD and PTD data among size classes, a Friedman repeated-measures non-parametric analysis of variance was undertaken in R. To account for repeated observations by individual fish, a unique tag identification number was included as a random variable. To determine whether the distribution of temperatures used at depth, relative to the SST (Δ*T*) varied among size classes, Kolmogorov–Smirnov tests were performed on the time-series data in R. To correct for multiple comparisons, a Bonferroni correction was applied to the *α* value.

The effect of environmental factors on the vertical habitat use of tagged *I. indica* was investigated using generalized additive mixed models (GAMMs). Two separate GAMMs were fitted to examine the relationship between environmental predictor variables and the response variables (MD and PTD <150 m) [24]. Environmental factors from the climatological databases, including Chl-*a*, SST, DO at 100 m depth intervals (DO_100–300 m_), mixed layer depth (MLD), sea-surface height deviation (SSHd), surface wind speed (Wind), location (longitude–latitude pair—derived from the most probable track) and month were modelled as fixed explanatory variables. GAMMs were constructed based on the same approach undertaken by Lam *et al.* [3], with analysis run using the package ‘mgcv’ in R and after visual assessment of the error distribution, a Gaussian family with an identity link function was applied [25]. All explanatory variables were modelled as continuous variables and smoothed, with the exception of month as it was categorical. To account for multiple observations from the same tagged fish, each fish was modelled as a random variable. Correlations between variables were investigated, and the presence of colinearity between covariates was assessed against the variance inflation factor (VIF) using the corvif function by Zuur *et al.* [26]. Variables were determined to be colinear if they contained VIF scores greater than 5 [26]. If colinearity was identified, the variables with the highest VIF values were sequentially removed until all VIF values were less than the threshold [26]. The initial, full factorial models were:

$$\begin{array}{rl} R_{i} & \;=s(\text{Location})+s(\text{MLD})+s(\text{SST})+s(\text{Wind})+s(\text{Chl - }a)+s(\text{SSHd})+s(\text{D}{\text{O}}_{100\;m})+s(\text{D}{\text{O}}_{200\;m})\\ & \;\;+s(\text{D}{\text{O}}_{300\;m})+\text{ Month }+\text{ Fis}{\text{h}}_{i}+\varepsilon , \end{array}$$

where *R_i_* is the response variable (MD or PDT) for Fish*_i_*, and *i* = 1,…, number of individual fish *n*; environmental predictor variables are defined earlier, *ϵ* is the Gaussian error term and smoothing functions were chosen automatically and evaluated manually using the ‘gam.check’ function. The amount of smoothing (*k*) applied to the splines was limited to ensure that the model did not overfit the data, yet sufficient to describe the nonlinearity between the response and explanatory variable [26].

The selection of an optimal model was undertaken using a backward selection strategy, whereby non-significant explanatory variables were dropped in sequential order until a final model containing only significant predictors was reached, as described by Lewis [24]. Statistical significance was set at *α* = 0.05 during the selection process. The final model was visually inspected to confirm the fit of all data and residuals in diagnostic plots. Explanatory variables were considered as strong predictors of vertical habitat use if they were significant in both PTD and MD models.

## 3. Results

Of the 102 PSATs analysed, 14 tags were physically recovered after pop-off. The number of *I. indica* tagged from each size class comprised: 14 small (20–50 kg), 16 intermediate (51–100 kg), 43 medium (101–250 kg), 14 large (251–400 kg) and 15 very large fish (greater than 400 kg). Tags stayed on animals for a median duration of 56 days (range 1–201) (electronic supplementary material, table S2). The displacement distance travelled by *I. indica* after tagging ranged from 10 to 10 623 km, including trans-oceanic and trans-equatorial movements. After filtering of erroneous tracks, a total of 6788 geolocation days were included in the final analysis.

### 3.1. Diving behaviour

Evaluation of time-series data resulted in the identification of two distinct diving behaviours: episodic deep-diving behaviour and bounce-diving behaviour (electronic supplementary material, figure S1). Episodic deep-diving behaviour was characterized by infrequent excursions below 400 m. These episodic deep dives were characterized by short dive durations (no longer than 20–30 min) and a ‘V’ shape dive profile, with animals spending less than 60 s at the maximum depth before ascending. Episodic deep-diving to depths greater than 400 m was observed in 28 individuals across all size classes. It was identified that episodic deep dives were more common in fish from adult size classes (greater than 50% of individuals from medium, large and very large classes) than in individuals from intermediate and small classes (less than 20%).

**Figure 1.**
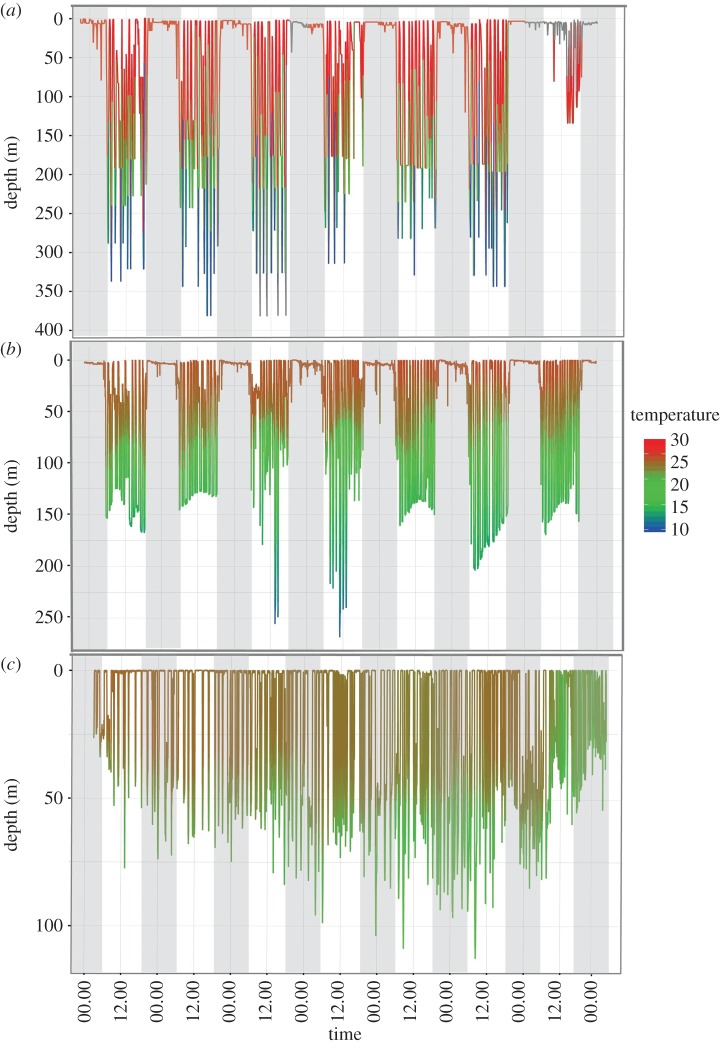
Typical week long dive profiles of varying sized *I. indica*: (*a*) dive profile of a 150 kg medium; (*b*) 90 kg intermediate; and (*c*) 30 kg small. Light shading identifies night-time hours (19.00–05.00 h) as defined by light-level data. All dive profiles were in waters off the continental shelf with a bathymetric depth greater than 1000 m.

Bounce-diving was characterized by fish remaining in the upper water column (approx. 20 m deep) during the night and undertaking a number of short repetitive dives between 80 and 400 m throughout the day (figure 1). The number of repetitive dives was often greater than 10 d^−1^, and bounce-diving behaviour appeared to start shortly after sunrise. Bounce-diving behaviour was found to be consistent among fish greater than 50 kg (intermediate, medium, large and very large size classes) with routine dives commencing at sunrise and occurring throughout the day until sunset. Although bounce-diving was detected in small fish, it appeared to be less frequent and occurred during both day and night (figure 1).

### 3.2. Vertical habitat use

**Figure 2.**
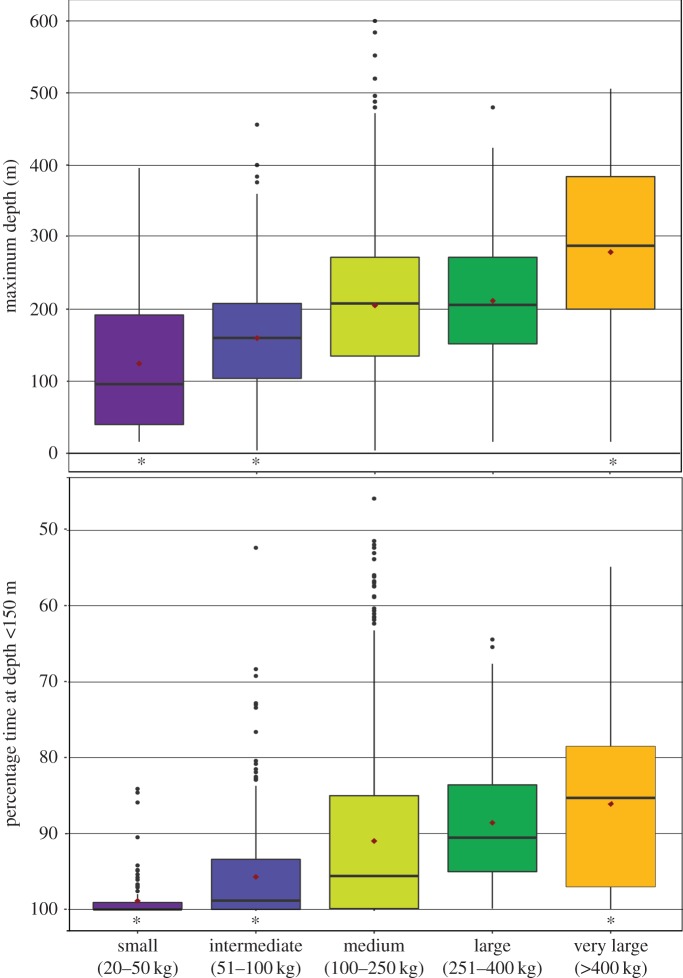
Influence of maturity stage on diving behaviour. Boxplots depicting the distribution of maximum diving depth (*a*) and percentage time at depth less than 150 m (*b*) across five size classes of *I. indica*. The box represents the first and third quartiles, the bold horizontal bar represents the median, black circles represent outliers, red diamonds indicate the mean and an asterisk indicates that a size class is significantly different from all other classes.

Tagged *I. indica* recorded a daily average MD of 206 m (±123 m), with 600 m being the deepest dive of any individual. Significant differences in the MD within the sub-sampled area of spatial overlap were detected among all five size classes (Friedman's test: *χ*^2^ = 60.05, *p* < 0.05), with the exception of between medium and large *I. indica* (figure 2). Overall, the mean MD increased with increasing size. A similar pattern of increasing depth of vertical habitat use was also observed in the PTD, with significant differences observed among all size classes, except for medium and large size classes (Friedman's test: *χ*^2^ = 49.08, *p* < 0.05). Large and very large fish spent greater than 10% of time below 150 m, contrasting with small and intermediate marlin which spent as little as 1.5% and 4.3% of time below 150 m, respectively.

### 3.3. Temperature use

**Figure 3.**
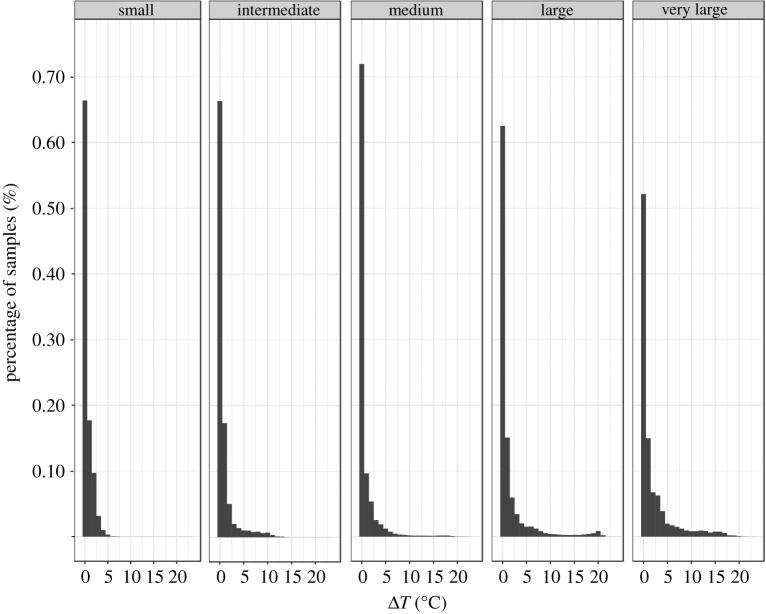
Temperature at depth use of *I. indica*.

*Istiompax indica* used a wide range of water temperatures, from 7.4°C to 31.3°C, although there was a higher occupancy of waters around 25–27°C. The frequency of temperature at depth distribution experienced by *I. indica* relative to the SST (Δ*T*) was significantly different among size classes (Kolmogorov–Smirnov test, *p* < 0.01). The range of Δ*T* values experienced by the various size classes varied from approximately 6.5°C in small to approximately 21.5°C in medium, large and very large *I. indica*. Despite differences in the magnitude of Δ*T* that were exhibited by different size classes, the majority of temperature records for all size classes were within 5°C of SST, amounting to approximately 87% for very large, 91% for large and intermediate, 97% for medium and approximately 99% for small size classes (figure 3).

### 3.4. Diel patterns

**Figure 4.**
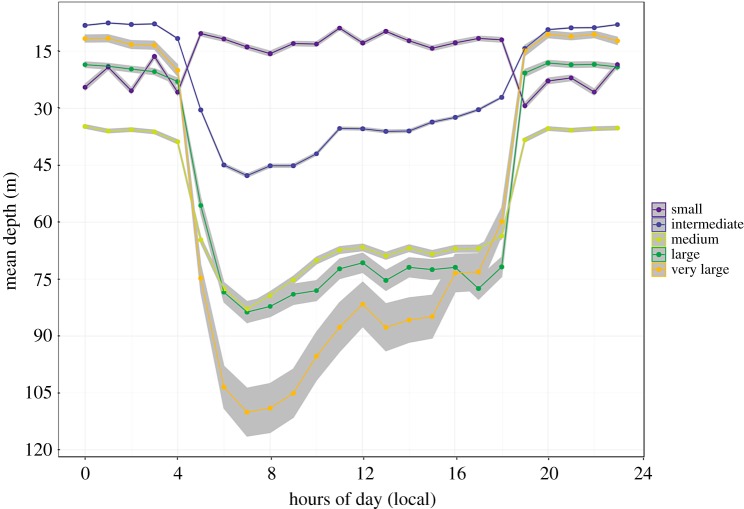
Diel behaviour of *I. indica*. Mean swimming depth over the course of a 24 h day for the five different size classes with 95% confidence intervals indicated by grey shading.

Pronounced diel patterns in vertical habitat use were detected for all individuals; however, they were not consistent across all size classes (figure 4). Occupancy of deeper depths during daylight hours was found to increase with size class. All size classes greater than 50 kg (intermediate–very large) occupied greater depths during the day than during the night. This increase in depth use during daylight hours was driven by the onset of bounce-diving, which resulted in movement into deeper waters (figure 1). During the night, bounce-diving ceased and animals larger than 50 kg were found to shift their behaviour to occupy surface waters (figure 4). By contrast, tagged *I. indica* from the smallest size class occupied shallower depth ranges during daylight hours than they did at night. The shift in vertical habitat use between day and night was also less pronounced than that observed in the larger size classes.

### 3.5. Factors influencing vertical habitat use

The effects of spatio-temporal and environmental factors on the daily vertical habitat use of *I. indica* were examined using two GAMMs. DO at 100 m, MLD, SSHd and location were all identified to significantly affect the PTD less than 150 m and MD (table 1). The best-fit model for PTD also included month and DO at 200 and 300 m as significant predictors. SST was found to be the only other variable significantly related to MD, whereas Chl-*a* and wind were not found to be related to either of the response variables. The best-fit models for both MD and PTD explained (adjusted *R*^2^) approximately 24% of the overall variability. Colinearity was not detected among any of the significant predictors in the MD or PTD models.

**Table 1.** Summary of GAMM results on vertical habitat use of *I. indica*. (Explanatory variables that are significant predictors in a model are indicated with a tick mark and grey shading identifies a significant predictor in both models (i.e. strong predictors).)

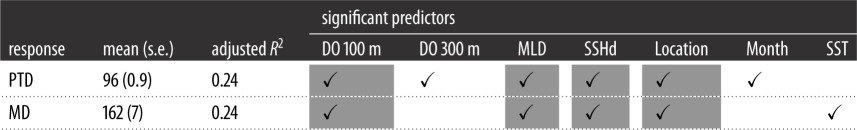

**Figure 5.**
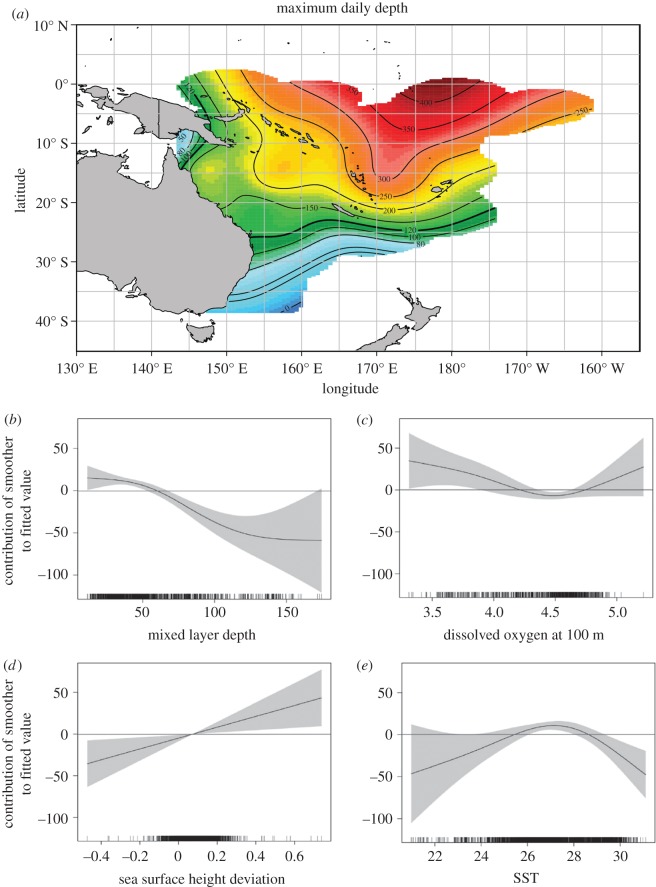
(*a*) Maximum daily depth by *I. indica*. Tag observations are binned to generate average values in a 1° × 1° grid and plotted in false colour (map). (*b–e*) Estimated individual effects (solid line) of environmental covariates on the maximum depth. Shaded areas show 95% confidence limits. Ticks on *x*-axis denote values for which there are data. To aid visualization, a horizontal line is added at 0 on the *y*-axis. Positive values on *y*-axis mean higher percentage.

**Figure 6.**
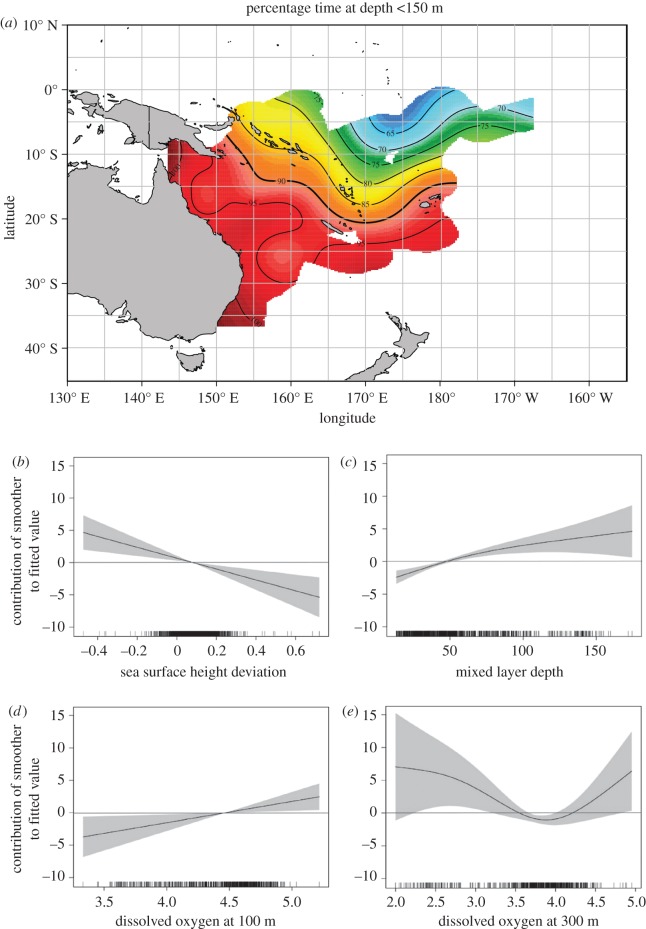
(*a*) Percentage time-at-depth less than 150 m by *I. indica*. Tag observations are binned to generate average values in a 1° × 1° grid and plotted in false colour (map). (*b–e*) Estimated individual effects (solid line) of environmental covariates on the percentage time at depth less than 150 m. Shaded areas show 95% confidence limits. Ticks on *x*-axis denote values for which there are data. To aid visualization, a horizontal line is added at 0 on the *y*-axis. Positive values on *y*-axis mean higher percentage.

The magnitude of the response of both MD and PTD changed when the MLDs were greater than 50 m. The MD increased when the SST was between 26°C and 28°C (figure 5). The spatial depth use appeared to vary on both a latitudinal and longitudinal gradient. The MD was found to increase notably as tagged animals occupied tropical waters to the north of the Tropic of Capricorn (approx. 23° S) and east of the Coral Sea basin (approx. 155° E). A similar spatial trend was noted in the percentage time at depth, where time in the upper 150 m of the water column decreased as fish moved to the northeast into warm tropical waters (figure 6). After fish left the Coral Sea basin, the PTD gradually decreased from approximately 90% to approximately 65%; however, the spatial trend was not as strong across a longitudinal gradient as seen for the MD.

A linear trend was observed for the MD and PTD in response to changes in the SSHd. The maximum daily depths recorded for *I. indica* became deeper with increases in the SSHd. As the PTD showed a negative response to increasing SSHd, it suggests that less time spent in the upper 150 m water column occurred with rising SSHd (figure 6). Decreases in the time spent in the upper water column by *I. indica* were also identified to occur when the availability of DO at 100 m improved. The response of DO_100 m_ to changing MD was found to be a nonlinear trend that was weak in magnitude (figure 5). This nonlinear response of MD to DO_100 m_ illustrated that greater maximum depths were observed when DO concentrations were less than 4.2 ml O_2_ l^−1^ or greater than 4.8 ml O_2_ l^−1^. A nonlinear trend was also noted in the response of DO_300 m_ to PTD, with the highest percentage of time spent in waters deeper than 150 m occurring when DO_300 m_ concentrations were between 3.5 and 4.2 ml O_2_ l^−1^.

## 4. Discussion

Accessing the world's largest PSAT tagging datasets on *I. indica* enabled us to undertake the most comprehensive analyses of the species' vertical habitat use and to examine differences in diving behaviour by size. In this study, we found that vertical habitat use expanded with increases in the mass of tagged animals. We also demonstrated the influence of environmental factors (including the MLD, DO and SSHa) and the spatial distribution on the maximum diving depth and time at depth. By characterizing the vertical habitat use of *I. indica* across the full-size range of the species, our investigation provides a foundation for exploring ontogenetic differences in other large pelagic predators.

### 4.1. Vertical habitat characterization

Vertical habitat use differed from that of previous satellite tagging investigations of *I. indica* [15–17]. Tagged animals in our study were observed to regularly access mesopelagic waters (greater than 200 m deep), with pronounced diel patterns in the diving behaviour, which had not previously been observed in *I. indica*. Our findings contrast with other studies in the South China Sea [15] and the southwestern Pacific Ocean [16], which concluded that *I. indica* was restricted to approximately 250 m of the upper water column, with individuals rarely diving below the mixed layer [15–17]. These observed differences in vertical habitat use are likely in part owing to regional differences, with fish tagged in the South China Sea exposed to differing environmental conditions and the collection of depth data at 15 min intervals in that study which may have precluded the detection of fine-scale diving behaviour (i.e. bounce-diving) [15]. By comparison, the analysis of *I. indica* diving behaviour by Gunn *et al.* [16] was limited to only two dive profiles (mean duration = 51 days), resulting in modest temporal and spatial coverage, which may have restricted the ability to capture the true extent of diving behaviour over longer time and area scales. However, the results reported here are similar to the diving patterns reported for many other large pelagic fish species including blue marlin (*M. nigricans*) [1], yellowfin tuna (*Thunnus albacares*) [27] and tiger sharks (*Galeocerdo cuvier*) [28], indicating that the vertical habitat use observed by *I. indica* is not uncommon among large pelagic predators.

The dive profiles of *I. indica* were characterized by two types of diving behaviour, which were similar to those observed for *T. albacares*, skipjack (*Katsuwonus pelamis*) and bigeye tuna (*Thunnus obesus*) [29], opportunistic deep-diving and bounce-diving. Opportunistic deep dives by *I. indica* were characterized by infrequent and unpredictable vertical movements to below 250 m, the deepest of which was recorded to 600 m in the Coral Sea. In striped marlin (*K. audax*), opportunistic deep-diving has been suggested as an act of predator avoidance owing to its irregular nature [3], and it is possible that opportunistic deep-diving of *I. indica* may also reflect an act of predator avoidance. Predation of five tagged fish was observed in this study, suggesting the relevance of predator avoidance as a potential motive for opportunistic deep-diving. Although opportunistic deep-diving was useful in defining the vertical limits of *I. indica*, this behaviour was infrequent and is not considered representative of normal vertical habitat use.

### 4.2. Ontogenetic variability

The depth exhibited by *I. indica* during bounce-diving behaviour varied considerably among size classes, but its commonality in occurrence indicated that it probably reflects an important behaviour. Bounce-diving has been extensively studied in several species of tuna and is suggested as a tactic to optimize time at depth when foraging [29]. The use of bounce-dives enables an individual to regularly access warm surface waters to raise body temperature between dives, which then allows for an increase in the time available to forage below the thermocline [28]. This bounce-dive behaviour in many large pelagic fishes is generally restricted to daylight hours when sunlight penetrates deep into the water column, facilitating the hunting ability of these large visual predators [3]. Recent evidence from dietary investigations provides support for foraging as a motive for *I. indica* to undertake bounce-diving to mesopelagic waters. Stomach content analysis has shown that *I. indica* fed almost exclusively on the purple-back flying squid (*Sthenoteuthis oualaniensis*), a deep-water species, during the day in the eastern Arabian Sea [30]. In the southwestern Pacific, the vertical distribution of *S. oualaniensis* closely correlates with the reported depth use of *I. indica* when bounce-diving [31], suggesting a causal link between diving behaviour and prey distribution.

Despite the importance of bounce-diving as a foraging behaviour, considerable differences in depth use were noted among size classes, which appeared to be reflected in the periodicity, frequency and depth of bounce-diving behaviour. Small *I. indica* were the only size class observed to undertake bounce-diving during the night. This was further highlighted by the small size class being the only one that exhibited a greater surface affinity during the day than at night. The repetitive-diving behaviour of small *I. indica* was often only to depths of 50–70 m, suggesting that if prey distribution is driving bounce-diving depth, then these smaller sized animals are likely to be targeting different prey sources than larger marlin. Gut content analysis of juvenile *I. indica* caught in the southwestern Pacific supports this theory, with small pelagic baitfishes (*Amblygaster sirm* and *Sardinella gibbosa*) identified as the dominant prey species of juveniles 10–40 kg [32]. Both *A. sirm* and *S. gibbosa* occupy neritic waters (less than 40 m deep), suggesting that differences in diurnal patterns and depth use may well reflect an ontogenetic diet shift [33].

Differences in vertical habitat use by animals of different size classes have also been reported for other pelagic predators. For example, ontogenetic niche expansions observed in the depth distribution of small and medium sized tiger sharks (*G. cuvier*) were attributed to both a dietary shift and increased thermal inertia in larger sharks [34]. It was also observed that young of the year *G. cuvier* used neritic habitats to enhance growth before presumably shifting to oceanic habitats and food sources [34]. This may be similar for small *I. indica*, whose use of the water column may also be in part owing to their occupancy of neritic waters [21]. As a result, depth use by *I. indica* and their prey species may be physically restricted by the shallow benthos of coastal waters. In the light of this, in this study, it was observed that when small fish occupied waters off the continental shelf (greater than 2000 m), they were seldom observed to dive to below 100 m (figure 1). This indicates that while prey distribution is likely to provide a motive for ontogenetic niche expansion, in deeper waters it is likely that environmental factors play a role in limiting the extent of vertical habitat use.

### 4.3. Physical drivers of depth use

Temperature influences the distribution and development of marine finfish, with preferred temperature ranges often coinciding with optimal growth rates [12,35]. For any fish species, optimal performance (e.g. oxygen uptake, heart rate and stroke volume, skeletal muscle contractility and cellular processes) is dependent on body temperature. For ectothermic species, as water temperature falls below their particular optimum operating temperature, they become physiologically compromised, which affects swimming performance [36]. Therefore, it is not surprising that temperature has been identified as one of the primary physical factors associated with depth use of billfishes [2,13]. The physiological effects of temperate also appears to influence vertical habitat use of *I. indica*, as observed in this study, where increases in depth use were associated with SST and MLD, suggesting an expansion of the thermal niche for larger individuals. The effect of temperature on *I. indica* was similar to that observed in *T. albacares*, where greater time spent at depths by larger fish was accredited to their enhanced physiological tolerance to low temperatures and DO concentrations [37]. In the current study, the maximum diving depth and time-at-depth increased as *I. indica* moved into warmer equatorial waters, consistent with the effects of temperature on performance [38].

It has been suggested that the temperature of the heart may play an important role in limiting the vertical movement of tunas and billfish [39]. In Pacific bluefin tuna (*Thunnus orientalis*), the effect of prolonged exposure of the heart to cold temperatures has been shown to drive collapse of cardiac output [36]. This may also be the case for istiophorid billfishes, as like tunas, the heart in marlin consists of a high-pressure, high stroke rate pump [40]. The heart in *M. nigricans* comprises about 0.15% of the body mass [40] and is relatively large compared with most other fishes. Marlin have well-developed coronary circulation which is unusual among teleost fishes, but common in other teleosts capable of sustained, fast swimming [41]. The coronary arteries arise from efferent branchial arteries (cranial source) and the dorsal aorta (caudal source), and deliver oxygenated blood to the heart. This blood, having recently passed through the gills, which are efficient heat exchangers, will be close to ambient water temperature [42,43]. Therefore, as marlin descend into cooler waters, there will be a rapid cooling of the heart that will affect cardiac performance, which is likely to be size-independent. Therefore, if the temperature of a marlin's heart was the limiting factor, it would be expected that the diving of all *I. indica* size classes would be restricted to a similar depth. Our finding is in contrast to this, as vertical habitat use (temperature range and depth range) expanded with body mass of tagged animals. As a result, if *I. indica* vertical habitat use is restricted by temperature, then it is likely that other aspects of their physiology, such as skeletal muscle contractile performance or lactic acid build-up in the tissues, represent the limiting factor [38]. Alternatively, it could be that marlin-diving behaviour is dictated by ecological factors such as prey availability and that it is not necessary for small marlin to access the colder mesopelagic waters as sufficient prey is available in the upper photic zone. However, in the absence of controlled data on the effect of temperature on istiophorid billfish cardiac (and skeletal) muscle, the physiological response of billfish to environmental change is restricted to that which may be surmised from tagging studies.

As with temperature, DO in surrounding water is a physical factor critical for maintaining physiological function in teleost fishes [11,22]. Despite billfishes having specialized gills for optimizing gas exchange, oxygen availability is still likely to affect their diving physiology owing to the high metabolic demands of being a large very active pelagic predator [44,45]. Tagging studies on istiophorids off the tropical eastern Pacific have shown that hypoxic waters are associated with a compression of the vertical habitat used by *I. platypterus* and *M. nigricans* [2,5]. Our findings also identified DO as a strong predictor of *I. indica* vertical habitat use in the southwestern Pacific. The presence of oxygen-rich waters in the southwestern Pacific and interaction between physical factors on the physiological response probably contributed to the nonlinear relationship between MD and DO_100 m_. In the central Pacific where the water column is also characterized by high oxygen availability, changes in DO have been found to influence the movement of tagged *M. nigricans* when fish were already physiologically stressed by operating outside their optimal thermal window [22]. Thus, as oxygen availability and temperature both decrease with increasing depth, this combination is likely to result in physiological stresses that ultimately limit available vertical habitat [22,46].

Understanding the effects of physical factors on the depth use of pelagic fishes is imperative given recent declines in the DO content of oceans because of global warming [47]. The equatorial Pacific Ocean has been associated with the largest declines in oxygen content of any oceanic region [47]. This decline, combined with associated changes in water temperatures, could drive changes in both the vertical and horizontal distribution of *I. indica* stocks regularly accessing these waters [47]. Along the east coast of Australia, the effects of ocean warming have reportedly shifted the preferred surface habitat of juvenile *I. indica* southward by approximately 88 km decade^−1^ and as much as approximately 300 km during El Nino years [9]. While considerable uncertainty remains as to how billfishes will respond to environmental change, changes to the migration phenology, spawning, vertical distribution and survival rate of larvae have all been suggested [48]. If pelagic species are unable to adapt to environmental change, it could also have broad-scale implications for fisheries, including a shift in global fishing effort, increased susceptibility to fishing gear (i.e. longline and gillnets) and even collapse of some fisheries [49].

In addition to the effects of temperature and oxygen availability, the other physical factor found to be a strong predictor of *I. indica* vertical habitat use was SSHd. Changes in SSH indicate the presence of mesoscale ocean features, resulting in SSHd increases often being associated with fronts or eddies [50]. Consequently, SSH is commonly investigated as a predictive variable for horizontal movement in billfish and has been linked with shifting species distributions and noted increases in CPUE [9,10]. The recent identification of SSH as a significant variable in depth use models for *L. guttatus* and *K. audax* suggests that cyclonic eddy systems may also play an important role in determining how large pelagic predators use the water column [3,8]. However, this is not surprising as increased levels of primary productivity during the formation of eddies drive the aggregation of popular prey items [50]. The association of SSH anomalies with changes in vertical habitat use may therefore be reflective of feeding behaviour during periods of high prey availability.

## 5. Conclusion and recommendations

Advancements in the field of wildlife telemetry have led to a growing number of investigations on the vertical habitat use of pelagic fishes [2]. Our results add new insights to this knowledge base by revealing fundamental differences in the vertical habitat use of different sized *I. indica*. Future investigations of intraspecific size differences in other species of billfish will be important to identify whether this relationship exists across the broader species group or varies among species. Furthermore, the differences in diving behaviour of *I. indica* observed between regions highlight the need for tagging in other areas such as the eastern Indian Ocean, to assess whether depth use is variable among *I. indica* populations there [51]. The findings here also enhance our understanding of the spatio-temporal and environmental factors that influence diving behaviour of istiophorid fishes. With physical factors that are associated with diving behaviour now well characterized in wild billfishes [2], there is a need for controlled swimming tunnel studies and the implanting of archival tags with internal temperature logging capabilities to further our understanding of how billfish respond to environmental change.

## Supplementary Material

Table S1

## Supplementary Material

Table S2

## Supplementary Material

Figure S1.

## Data accessibility

The data accompanying this manuscript are available through the Dryad digital repository: http://dx.doi.org/10.5061/dryad.4nt0d [52]. Additional data from the *I. indica* tagged as part of the GMR tagging programme can also be publicly accessed through the Animal Telemetry Network website (http://oceanview.pfeg.noaa.gov/ATN/#).
