# Supplementary material for: Environmental influences and ontogenetic differences in vertical habitat use of black marlin (Istiompax indica) in the southwestern Pacific
Source: R Soc Open Sci. 2017 Nov 1;4(11):170694. doi: 10.1098/rsos.170694 (PMC5717634; doi:10.1098/rsos.170694)
Supplement: Table S1 [file rsos170694supp1.docx]

Table S1. Meta data summary of environmental data sources and intervals.

| **Variable** | **Interval** | **Database** |
| --- | --- | --- |
| Sea surface Temperature | daily | SST, Daily Optimum Interpolation |
| Wind | daily | NOAA/NCDC Blended Daily 0.25-degree Sea Surface Winds |
| Sea Surface Height Deviation | daily | Sea Surface Height Deviation, Aviso, Science Quality |
| Mixed Layer Depth | monthly | GODAS: Global Ocean Data Assimilation System |
| Dissolved at Oxygen 100 m | monthly | World Ocean Atlas 2009, Monthly Climatology, 1 degree, Temperature, Salinity, Oxygen |
| Dissolved at Oxygen 200 m | monthly | World Ocean Atlas 2009, Monthly Climatology, 1 degree, Temperature, Salinity, Oxygen |
| Dissolved at Oxygen 300 m | monthly | World Ocean Atlas 2009, Monthly Climatology, 1 degree, Temperature, Salinity, Oxygen |
| Chlorophyll-a | fortnight | Chlorophyll-a, Aqua MODIS, NPP |
