# Supplementary material for: Environmental influences and ontogenetic differences in vertical habitat use of black marlin (Istiompax indica) in the southwestern Pacific
Source: R Soc Open Sci. 2017 Nov 1;4(11):170694. doi: 10.1098/rsos.170694 (PMC5717634; doi:10.1098/rsos.170694)
Supplement: Table S2 [file rsos170694supp2.docx]

Table S2. Details for setup, release, and metadata from 102 PSATs deployed on black marlin.

| **Tag #** | **Date tagged** | **Duration (days)** | **Displacement Distance (km)** | **Size (kg)** | **Size Class** | **Sampling Interval (s)** | **Maximum Depth (m)** | **Program** | **Tag Manufacturer** | **Time-Series Data** |
| --- | --- | --- | --- | --- | --- | --- | --- | --- | --- | --- |
| 1 | 11/10/2002 | 8 | 13 | 180 | Medium | 20 | 176 | Domeier & Speare 2012 | WC | yes |
| 2 | 26/09/2003 | 10 | 137 | 68 | Intermediate | 20 | 456 | Domeier & Speare 2012 | WC | yes |
| 3 | 30/09/2003 | 9 | 68 | 180 | Medium | 20 | 136 | Domeier & Speare 2012 | WC | no |
| 4 | 30/09/2003 | 34 | 70 | 135 | Medium | 20 | 232 | Domeier & Speare 2012 | WC | no |
| 5 | 30/09/2003 | 79 | 264 | 225 | Medium | 20 | 368 | Domeier & Speare 2012 | WC | no |
| 6 | 30/09/2003 | 98 | 894 | 90 | Intermediate | 20 | 384 | Domeier & Speare 2012 | WC | no |
| 7 | 1/10/2003 | 136 | 720 | 124 | Medium | 20 | 536 | Domeier & Speare 2012 | WC | yes |
| 8 | 3/10/2003 | 12 | 100 | 90 | Intermediate | 20 | 232 | Domeier & Speare 2012 | WC | no |
| 9 | 4/10/2003 | 105 | 1445 | 124 | Medium | 20 | 348 | Domeier & Speare 2012 | WC | no |
| 10 | 8/10/2003 | 11 | 85 | 135 | Medium | 20 | 296 | Domeier & Speare 2012 | WC | no |
| 11 | 8/10/2003 | 101 | 1494 | 180 | Medium | 20 | 344 | Domeier & Speare 2012 | WC | no |
| 12 | 9/10/2003 | 82 | 827 | 135 | Medium | 20 | 248 | Domeier & Speare 2012 | WC | no |
| 13 | 10/10/2003 | 31 | 53 | 135 | Medium | 20 | 160 | Domeier & Speare 2012 | WC | no |
| 14 | 10/10/2003 | 85 | 1019 | 59 | Intermediate | 20 | 288 | Domeier & Speare 2012 | WC | no |
| 15 | 13/10/2003 | 82 | 1085 | 135 | Medium | 20 | 376 | Domeier & Speare 2012 | WC | yes |
| 16 | 14/10/2003 | 70 | 2015 | 180 | Medium | 20 | 276 | Domeier & Speare 2012 | WC | no |
| 17 | 15/10/2003 | 71 | 1400 | 248 | Medium | 20 | 396 | Domeier & Speare 2012 | WC | no |
| 18 | 16/10/2003 | 2 | 14 | 405 | Very Large | 20 | 24 | Domeier & Speare 2012 | WC | yes |
| 19 | 17/10/2003 | 120 | 1915 | 405 | Very Large | 20 | 356 | Domeier & Speare 2012 | WC | no |
| 20 | 17/10/2003 | 98 | 2723 | 180 | Medium | 20 | 468 | Domeier & Speare 2012 | WC | no |
| 21 | 27/10/2003 | 9 | 124 | 270 | Large | 20 | 232 | Domeier & Speare 2012 | WC | no |
| 22 | 27/10/2003 | 68 | 1866 | 203 | Medium | 20 | 448 | Domeier & Speare 2012 | WC | no |
| 23 | 28/10/2003 | 77 | 4369 | 450 | Very Large | 20 | 244 | Domeier & Speare 2012 | WC | no |
| 24 | 14/11/2003 | 51 | 3732 | 360 | Large | 20 | 384 | Domeier & Speare 2012 | WC | no |
| 25 | 15/11/2003 | 3 | 157 | 203 | Medium | 20 | 128 | Domeier & Speare 2012 | WC | no |
| 26 | 22/10/2004 | 31 | 189 | 25 | Small | 20 | 38 | Domeier & Speare 2012 | MT | yes |
| 27 | 16/11/2004 | 56 | 253 | 20 | Small | 20 | 80 | Domeier & Speare 2012 | MT | yes |
| 28 | 1/03/2005 | 13 | 653 | 59 | Intermediate | 20 | 40 | Domeier & Speare 2012 | WC | no |
| 29 | 5/03/2005 | 122 | 1115 | 30 | Small | 20 | 300 | Domeier & Speare 2012 | WC | no |
| 30 | 12/03/2005 | 183 | 1356 | 27 | Small | 20 | 344 | Domeier & Speare 2012 | WC | no |
| 31 | 20/03/2005 | 56 | 1170 | 35 | Small | 20 | 225 | Domeier & Speare 2012 | WC | no |
| 32 | 20/03/2005 | 6 | 39 | 30 | Small | 20 | 72 | Domeier & Speare 2012 | WC | yes |
| 33 | 7/04/2005 | 1 | 32 | 27 | Small | 20 | 16 | Domeier & Speare 2012 | WC | no |
| 34 | 30/11/2005 | 3 | 144 | 225 | Medium | 20 | 80 | Domeier & Speare 2012 | WC | no |
| 35 | 25/02/2006 | 47 | 469 | 69 | Intermediate | 20 | 270 | Domeier & Speare 2012 | WC | no |
| 36 | 26/02/2006 | 6 | 158 | 60 | Intermediate | 20 | 140 | Domeier & Speare 2012 | WC | no |
| 37 | 26/02/2006 | 9 | 81 | 60 | Intermediate | 20 | 144 | Domeier & Speare 2012 | WC | no |
| 38 | 23/08/2006 | 116 | 526 | 23 | Small | 20 | 80 | Domeier & Speare 2012 | WC | no |
| 39 | 5/10/2006 | 180 | 1773 | 68 | Intermediate | 20 | 456 | Domeier & Speare 2012 | WC | yes |
| 40 | 8/10/2006 | 8 | 314 | 113 | Medium | 20 | 112 | Domeier & Speare 2012 | WC | no |
| 41 | 15/10/2006 | 65 | 854 | 90 | Intermediate | 20 | 216 | Domeier & Speare 2012 | WC | yes |
| 42 | 16/10/2006 | 109 | 1552 | 68 | Intermediate | 20 | 344 | Domeier & Speare 2012 | WC | no |
| 43 | 3/11/2006 | 122 | 3711 | 158 | Medium | 20 | 488 | Domeier & Speare 2012 | WC | no |
| 44 | 4/11/2006 | 180 | 1341 | 135 | Medium | 20 | 225 | Domeier & Speare 2012 | WC | no |
| 45 | 6/11/2006 | 110 | 4955 | 158 | Medium | 20 | 400 | Domeier & Speare 2012 | WC | no |
| 46 | 6/11/2006 | 6 | 18 | 169 | Medium | 20 | 300 | Domeier & Speare 2012 | WC | no |
| 47 | 13/11/2006 | 5 | 48 | 135 | Medium | 20 | 300 | Domeier & Speare 2012 | WC | no |
| 48 | 27/11/2006 | 39 | 1384 | 113 | Medium | 20 | 360 | Domeier & Speare 2012 | WC | no |
| 49 | 30/11/2006 | 66 | 2267 | 180 | Medium | 20 | 328 | Domeier & Speare 2012 | WC | no |
| 50 | 30/11/2006 | 6 | 253 | 81 | Intermediate | 20 | 176 | Domeier & Speare 2012 | WC | yes |
| 51 | 21/02/2007 | 24 | 106 | 45 | Small | 20 | 184 | Domeier & Speare 2012 | WC | no |
| 52 | 21/02/2007 | 31 | 128 | 25 | Small | 20 | 184 | Domeier & Speare 2012 | WC | no |
| 53 | 25/02/2007 | 15 | 38 | 29 | Small | 20 | 232 | Domeier & Speare 2012 | WC | yes |
| 54 | 24/03/2007 | 46 | 1178 | 20 | Small | 20 | 216 | Domeier & Speare 2012 | WC | no |
| 55 | 24/03/2007 | 1 | 10 | 20 | Small | 20 | 48 | Domeier & Speare 2012 | WC | yes |
| 56 | 5/05/2007 | 37 | 0 | 84 | Intermediate | 20 | 244 | Domeier & Speare 2012 | WC | no |
| 57 | 3/11/2008 | 32 | 1381 | 360 | Large | 20 | 195 | Domeier & Speare 2012 | WC | no |
| 58 | 27/11/2008 | 9 | 295 | 293 | Large | 20 | 264 | Domeier & Speare 2012 | WC | no |
| 59 | 4/12/2008 | 64 | 1999 | 383 | Large | 20 | 552 | Domeier & Speare 2012 | WC | no |
| 60 | 21/10/2009 | 2 | 73 | 81 | Intermediate | 20 | 400 | Domeier & Speare 2012 | WC | no |
| 61 | 22/10/2009 | 27 | 64 | 135 | Medium | 20 | 216 | Domeier & Speare 2012 | WC | no |
| 62 | 4/10/2011 | 77 | 492 | 408 | Very Large | 60 | 496 | Great Marlin Race | WC | no |
| 63 | 5/10/2011 | 96 | 619 | 113 | Medium | 60 | 520 | Great Marlin Race | WC | no |
| 64 | 6/10/2011 | 120 | 4308 | 191 | Medium | 60 | 448 | Great Marlin Race | WC | no |
| 65 | 13/10/2011 | 120 | 501 | 91 | Intermediate | 60 | 400 | Great Marlin Race | WC | no |
| 66 | 16/10/2011 | 85 | 5072 | 408 | Very Large | 60 | 360 | Great Marlin Race | WC | no |
| 67 | 15/03/2012 | 54 | 739 | 136 | Medium | 60 | 312 | Great Marlin Race | WC | no |
| 68 | 26/10/2012 | 90 | 660 | 400 | Large | 60 | 312 | Great Marlin Race | WC | no |
| 69 | 2/11/2012 | 43 | 10 | 544 | Very Large | 60 | 568 | Great Marlin Race | WC | no |
| 70 | 5/11/2012 | 53 | 2522 | 500 | Very Large | 60 | 384 | Great Marlin Race | WC | no |
| 71 | 7/11/2012 | 29 | 170 | 200 | Medium | 60 | 432 | Great Marlin Race | WC | yes |
| 72 | 11/11/2012 | 68 | 4775 | 360 | Large | 60 | 376 | Great Marlin Race | WC | no |
| 73 | 15/10/2013 | 180 | 1091 | 136 | Medium | 60 | 496 | Great Marlin Race | WC | no |
| 74 | 17/10/2013 | 187 | 358 | 113 | Medium | 60 | 48 | Great Marlin Race | WC | no |
| 75 | 18/10/2013 | 120 | 807 | 181 | Medium | 60 | 480 | Great Marlin Race | WC | no |
| 76 | 19/10/2013 | 9 | 10 | 45 | Small | 60 | 176 | Great Marlin Race | WC | no |
| 77 | 21/10/2013 | 4 | 86 | 408 | Very Large | 60 | 112 | Great Marlin Race | WC | no |
| 78 | 22/10/2013 | 4 | 48 | 113 | Medium | 60 | 16 | Great Marlin Race | WC | no |
| 79 | 23/10/2013 | 36 | 278 | 363 | Large | 60 | 512 | Great Marlin Race | WC | no |
| 80 | 29/10/2013 | 172 | 2290 | 181 | Medium | 60 | 208 | Great Marlin Race | WC | no |
| 81 | 29/10/2013 | 7 | 47 | 227 | Medium | 60 | 64 | Great Marlin Race | WC | no |
| 82 | 24/11/2013 | 180 | 1671 | 91 | Intermediate | 60 | 408 | Great Marlin Race | WC | no |
| 83 | 12/10/2014 | 27 | 344 | 386 | Large | 60 | 192 | Great Marlin Race | WC | yes |
| 84 | 12/10/2014 | 180 | 3564 | 431 | Very Large | 60 | 408 | Great Marlin Race | WC | yes |
| 85 | 12/10/2014 | 5 | 45 | 386 | Large | 60 | 39 | Great Marlin Race | WC | yes |
| 86 | 13/10/2014 | 181 | 4611 | 454 | Very Large | 60 | 464 | Great Marlin Race | WC | yes |
| 87 | 13/10/2014 | 133 | 2918 | 450 | Very Large | 60 | 506 | Great Marlin Race | WC | yes |
| 88 | 13/10/2014 | 180 | 1631 | 408 | Very Large | 60 | 480 | Great Marlin Race | WC | yes |
| 89 | 13/10/2014 | 9 | 265 | 431 | Very Large | 60 | 152 | Great Marlin Race | WC | yes |
| 90 | 14/10/2014 | 108 | 5652 | 454 | Very Large | 60 | 424 | Great Marlin Race | WC | yes |
| 91 | 14/10/2014 | 9 | 158 | 159 | Medium | 60 | 160 | Great Marlin Race | WC | yes |
| 92 | 14/10/2014 | 201 | 1748 | 136 | Medium | 60 | 210 | Great Marlin Race | WC | yes |
| 93 | 15/10/2014 | 181 | 6844 | 200 | Medium | 60 | 584 | Great Marlin Race | WC | yes |
| 94 | 15/10/2014 | 183 | 1833 | 272 | Large | 60 | 496 | Great Marlin Race | WC | yes |
| 95 | 16/10/2014 | 16 | 366 | 250 | Medium | 60 | 600 | Great Marlin Race | WC | yes |
| 96 | 17/10/2014 | 180 | 7066 | 340 | Large | 60 | 480 | Great Marlin Race | WC | yes |
| 97 | 17/10/2014 | 180 | 1475 | 272 | Large | 60 | 600 | Great Marlin Race | WC | yes |
| 98 | 22/10/2014 | 180 | 1120 | 150 | Medium | 60 | 506 | Great Marlin Race | WC | yes |
| 99 | 23/10/2014 | 181 | 1840 | 113 | Medium | 60 | 352 | Great Marlin Race | WC | yes |
| 100 | 28/10/2014 | 180 | 10623 | 499 | Very Large | 60 | 472 | Great Marlin Race | WC | yes |
| 101 | 28/10/2014 | 13 | 142 | 181 | Medium | 60 | 272 | Great Marlin Race | WC | yes |
| 102 | 1/11/2014 | 10 | 10 | 318 | Large | 60 | 116 | Great Marlin Race | WC | yes |

Note: Tag manufacturers have been abbreviated as follows; Wildlife Computers (WC) and Microwave Telemetry (MT)
