# Supplementary material for: Environmental influences and ontogenetic differences in vertical habitat use of black marlin (Istiompax indica) in the southwestern Pacific
Source: R Soc Open Sci. 2017 Nov 1;4(11):170694. doi: 10.1098/rsos.170694 (PMC5717634; doi:10.1098/rsos.170694)
Supplement: Figure S1. [file rsos170694supp3.docx]

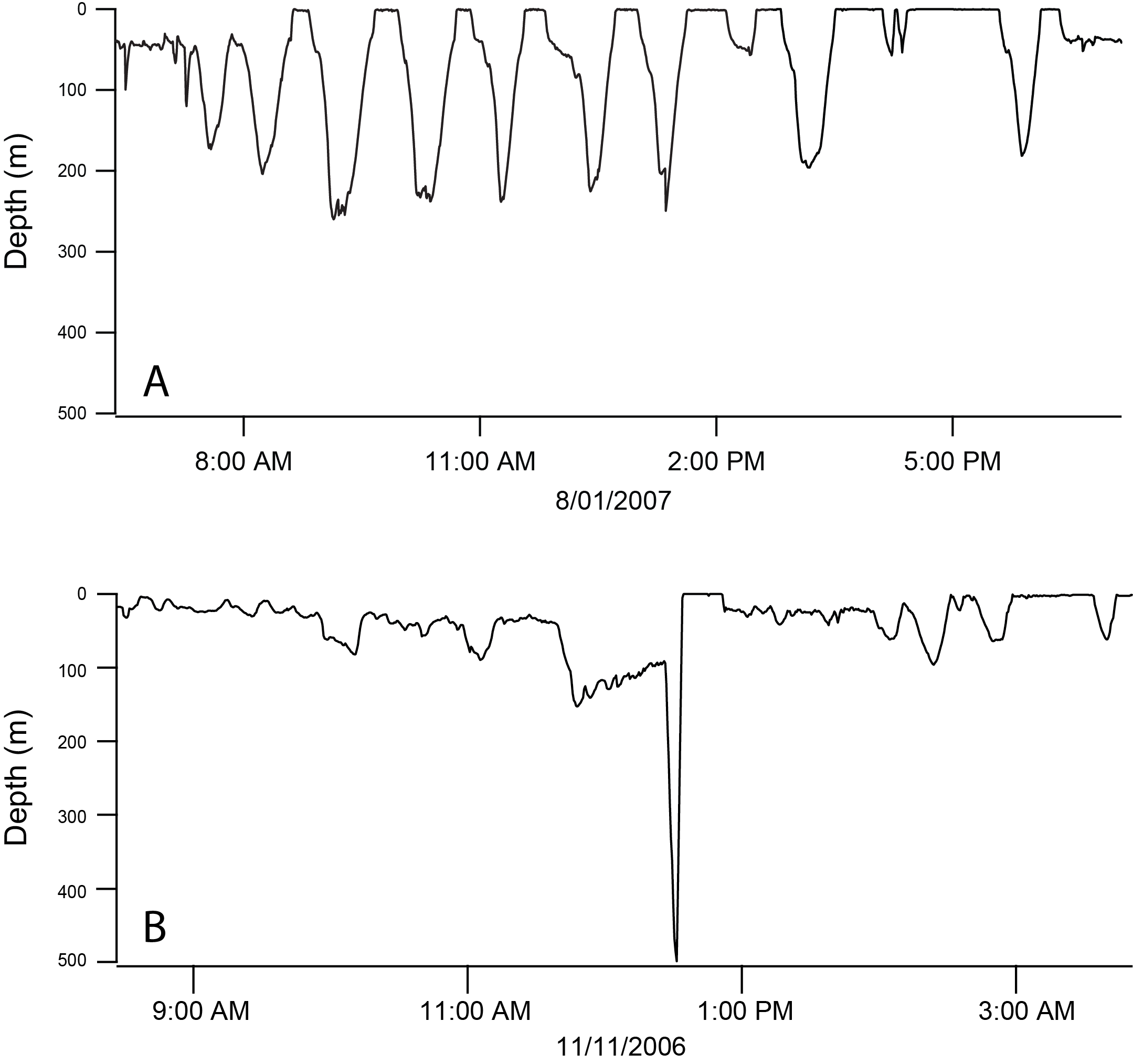


Figure S1. Dive profiles from a 68kg (Intermediate size class) black marlin demonstrating; A) bounce diving behaviour and B) episodic deep-diving behaviour.
